# Evaluating nomogram models for predicting survival outcomes in gastric gastrointestinal stromal tumors with SEER database analysis

**DOI:** 10.1038/s41598-024-62353-z

**Published:** 2024-05-20

**Authors:** Liuliang Yong, Lanjun Li, Jun Wu, Pan Liang, Jianbo Gao

**Affiliations:** 1 School of Electrical and Information Engineering, Zhengzhou University, Zhengzhou, 450001 China; 2 Department of Radiology, The First Affiliated Hospital of Zhengzhou University, Zhengzhou, 450052 China; 3 Department of Neurology, The First Affiliated Hospital of Zhengzhou University, Zhengzhou, 450052 China

**Keywords:** SEER, Gastric GISTs, Categorical variable, Cox regression model, Penalized technique, Gastroenterology, Gastrointestinal diseases, Risk factors

## Abstract

Gastrointestinal stromal tumors (GISTs) predominantly develop in the stomach. While nomogram offer tremendous therapeutic promise, there is yet no ideal nomogram comparison customized specifically for handling categorical data and model selection related gastric GISTs. (1) We selected 5463 patients with gastric GISTs from the SEER Research Plus database spanning from 2000 to 2020; (2) We proposed an advanced missing data imputation algorithm specifically designed for categorical variables; (3) We constructed five Cox nomogram models, each employing distinct methods for the selection and modeling of categorical variables, including Cox (Two-Stage), Lasso-Cox, Ridge-Cox, Elastic Net-Cox, and Cox With Lasso; (4) We conducted a comprehensive comparison of both overall survival (OS) and cancer-specific survival (CSS) tasks at six different time points; (5) To ensure robustness, we performed 50 randomized splits for each task, maintaining a 7:3 ratio between the training and test cohorts with no discernible statistical differences. Among the five models, the Cox (Two-Stage) nomogram contains the fewest features. Notably, at Near-term, Mid-term, and Long-term intervals, the Cox (Two-Stage) model attains the highest Area Under the Curve (AUC), top-1 ratio, and top-3 ratio in both OS and CSS tasks. For the prediction of survival in patients with gastric GISTs, the Cox (Two-Stage) nomogram stands as a simple, stable, and accurate predictive model with substantial promise for clinical application. To enhance the clinical utility and accessibility of our findings, we have deployed the nomogram model online, allowing healthcare professionals and researchers worldwide to access and utilize this predictive tool.

## Introduction

Gastrointestinal stromal tumors (GISTs) are the most prevalent mesenchymal tumors of the gastrointestinal (GI) tract, accounting for approximately 0.1–3% of all GI malignancies^1^. They can arise anywhere in the digestive tract, with the stomach being the most common site, comprising about 60–70% of all cases, followed by the small intestine. Less common sites include the esophagus, colon, rectum, and extragastrointestinal regions^2^. Surgery is the most prevalent therapeutic technique. While the prognosis for most GISTs patients following surgery is excellent, tumor recurrence is a typical occurrence in GISTs patients^3^. Regarding the postoperative survival progression of GISTs originating in the stomach and small intestine, some studies^4,5^ reveal no difference, while a recent study^6^ suggests a difference, therefore there is no conclusive consensus. Thus, postoperative determination of survival progression in patients with gastric GISTs remains clinically significant.

Due to the rarity of GISTs, big datasets such as the Surveillance, Epidemiology, and End Results (SEER) Program can serve as a global real-world cohort database for researching GISTs^7–11^. Since data missingness is a typical occurrence in real-world data gathering, some studies^7,8,10,11^ have categorized missing data as 'unknown' as one of the values for multi-category variables in subsequent study. However, such a basic method requires that the missingness of the 'unknown' is balanced across different categories, which is difficult to satisfy. On the other hand, several studies^12^ have adopted missing data imputation methods, however simple imputation can bring irreparable bias into the research^13^. Furthermore, the SEER database largely comprises multi-categorical variables with missing values, while most existing imputation approaches lack flexibility for multi-categorical variables.

Cox nomogram is a clinically informative modeling and visualization tool. Cox nomogram can integrate both independent components and composite indices. However, due to the inclusion of composite indices (such as the AJCC stage), which may cover some independent clinical markers (such as mitotic rate), a certain level of collinearity may occur. Directly adding them may lead to model non-convergence and instability. The traditional design of the cox nomogram normally involves a two-stage process of single-factor and multi-factor selection before nomogram construction^7–11^. However, with the widespread usage of Least Absolute Shrinkage and Selection Operator (LASSO) in clinical research as a feature selection and standalone model, Penalized Models have steadily developed as a unique clinical modeling strategy to replace the old two-stage method^14–17^. In the area of survival prediction for gastric GISTs, whether the Penalized Cox Regression Model^18^ can replace the usual two-stage modeling technique has not been compared in any study.

The unpredictability produced by the segmentation of train and test cohorts is an issue that is often disregarded and underestimated, however it can considerably contribute to the problem of irreproducible trials^19^. Studies have demonstrated the direct influence of data tampering on statistical outcomes, therefore creating an increasing emphasis on issues surrounding ‘p-hacking’ within the area of medical statistics^20^. While cross-validation stands as an excellent approach, it’s worth mentioning that in most studies, cross-validation is often implemented exclusively during the model parameter estimation phase, resulting in a single evaluation of the test dataset. A one-time separation of data into train and test cohorts, in the absence of an external validation dataset, introduces an undesired level of randomness.

In summary, this study intends to increase the design of a simpler and more stable postoperative survival prediction nomogram for gastric GISTs, and the changes made include the following:

1. Innovatively proposed the MissCatBoosts missing data technique for multicategorical variables;
2. The data for both OS and CSS completed 50 iterations of train/test cohort, guaranteeing that no statistically significant differences existed for each variable;
3. We conducted a detailed comparison of five nomogram models for both OS and CSS tasks across six-time points.

## Materials and methods

The recent ASCI text data version of the Surveillance, Epidemiology, and End Results (SEER) Program of the National Cancer Institute in the USA was the source of the present population-based analysis with permission to obtain research data from the latest SEER database (Approved account: 19047-Nov2021). Primary cancer location and histological features were coded according to criteria in the third version of the International Classification of Diseases for Oncology (ICD-O-3). This study was in light of the public usage of deidentified data from the SEER database and did not involve interaction with human individuals or the use of personal identifying information. Therefore, there is no need to require formal informed permission from the SEER recorded cases in the study.

### Data source and population selection

Imatinib mesylate was approved by the Food and Drug Administration (FDA) for the treatment of GISTs in the year 2002 after clinical trials demonstrated that its use postoperatively in intermediate- to high-risk patients prolonged cancer-specific survival (CSS) as well as overall survival (OS)^21^, so “Incidence-SEER Research Data,17 Registries, Nov 2022 Sub[2000-2020]” was selected by SEER*Stat 8.4.2 (https://seer.cancer.gov/) and GISTs patients were identified by codes “8936/3” for ICD-O-3 histology types with 14,745 patients.

**Figure 1:**
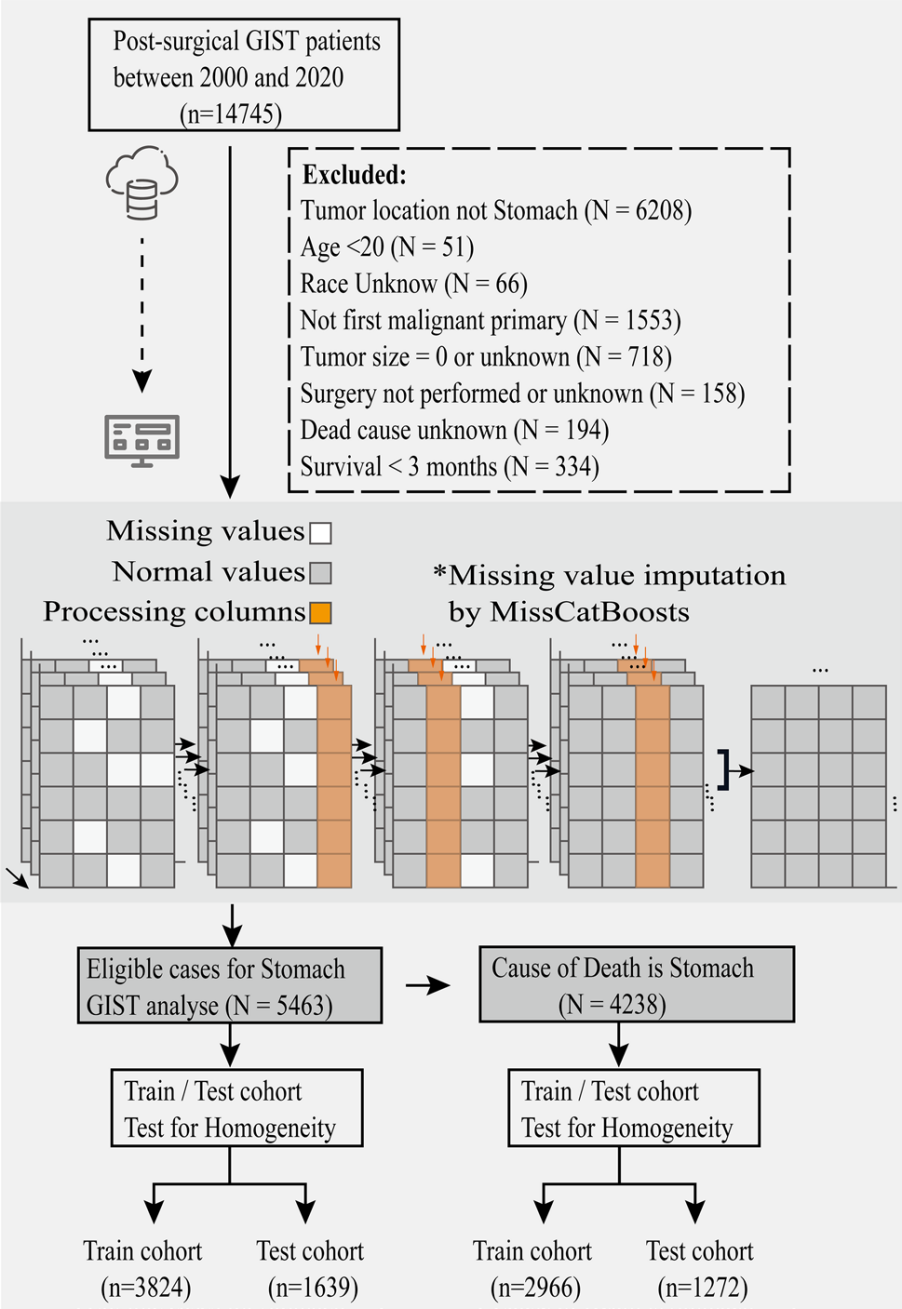
Flowchart of the screening and preprocessing procedure for patients with gastric GISTs.

The exclusion criteria include: (I) patients with site recode other than stomach; (II) patients with age recode less than 20; (III) patients with race recode unknown; (IV) patients without first malignant; (V) patients with tumor size unknown or 0; (VI) patients with surgency not performed or unknown; (VII) patients with dead cause unknown; (VIII) patients with survival months less than 3 months (Fig. 1).

For each patient, study characteristics were acquired including age at diagnosis, race, sex, marital status, tumor size, tumor grade, tumor site, AJCC stage, mitotic rate, surgical management, chemotherapy, survival months, and cause of death. Age at diagnosis was viewed as a numeric variable.

### Missing data

Missing values for multi-categorical variables were handled by MissCatBoosts multiple imputations (Fig. 1). To begin, we make an initial guess for the missing variables using mean imputation or another imputation approach. Then, arrange the variables according to the amount of missing values starting with the lowest amount. For each variable, the missing values are imputed by first training a CatBoost model with response and predictors; and then, predicting the missing values by applying the learned CatBoost model. The imputation technique is performed until a stopping requirement is fulfilled. At finally, the final imputed matrix was created by the aggregation of different imputations. The pseudo algorithm (Table S1) presents a representation of the MissCatBoosts approach.

### Statistical analysis

Statistical studies were conducted using Python 3.9.13, Numpy 1.24.3, Statsmodels 0.13.5, and R software (version 4.2.2). Based on the National Comprehensive Cancer Network (NCCN) guidelines Version 2.2023, we changed quantitative factors, such as tumor size, mitotic rate, and regional nodes inspected into categorical data. The tumor size was separated as ≤ 2, 2–5, 5–10, and ≥ 10 cm. The mitotic rate was separated as ≤ 5/5 and > 5/5 mm^2^ HPF. The regional nodes investigated were split as 0, 1–4, and > 4. We employed the Kaplan–Meier survival curve to examine the groups in OS and CSS, and the differences were assessed by applying the Log-rank test. Categorical variables are subjected to one-hot encoding and then dummy variable handling before being entered into the model. We apply the Phi coefficient to measure the association across categorical data, and visually show it through a heatmap. For nomogram creation and validation, patients in the SEER database were randomly sorted into train and test cohorts according to a ratio of 7: 3. Numeric variables were shown as median and interquartile range (IQR) in train and test cohorts and were evaluated using Kruskal–Wallis test. Categorical variables were shown as frequencies and proportions in train and test cohorts and were evaluated using the Chi­Squared test. Items identified as statistically significant in the univariate Cox regression analysis were applied to multivariate analysis utilizing a train cohort. Multivariate Cox regression analysis was used to individually examine the association of all factors with OS by computing hazard ratios and 95% CIs. Significant items (p < 0.05) were selected as the independent predictors. The train-test cohort splitting and significance test of the CSS referred to the OS task.

**Figure 2:**
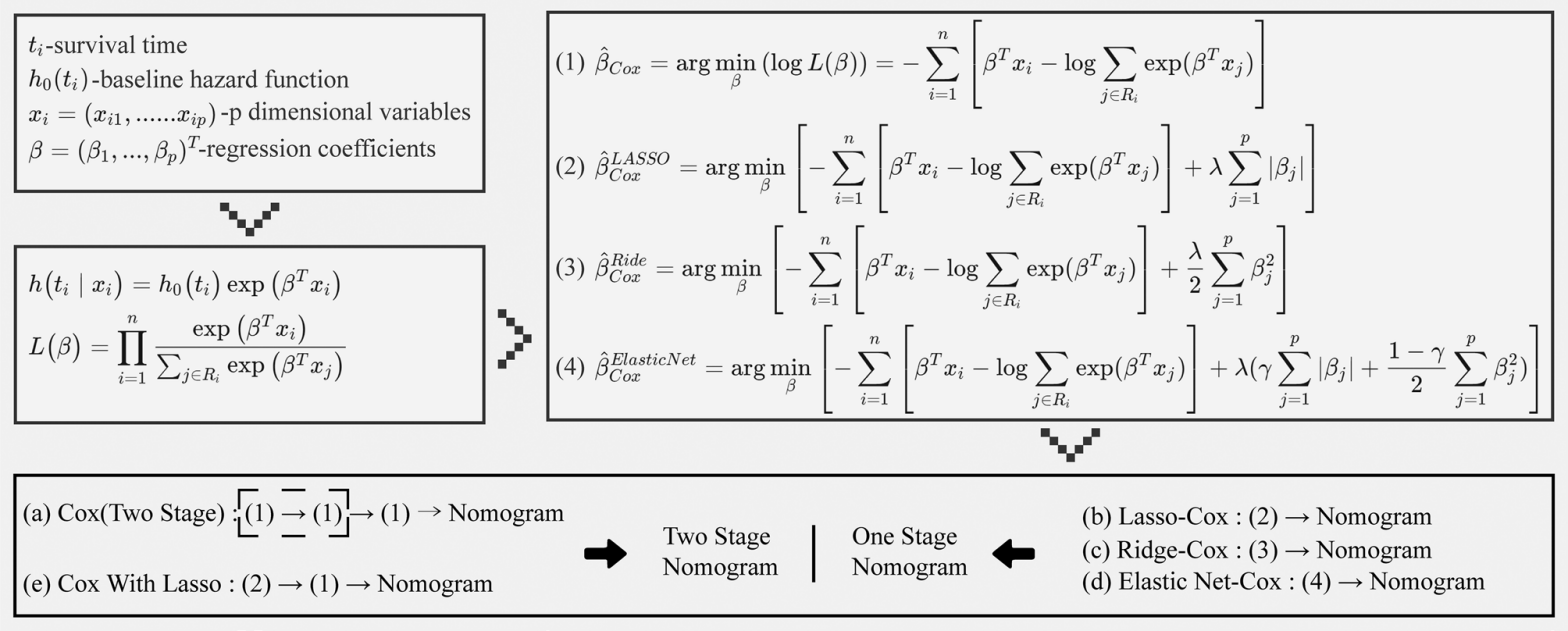
Flowchart for constructing five types of nomograms: (**a**) conducting univariate and multivariate feature selection using equation 1, followed by nomogram parameter determination using equation 1; (**b**) simultaneous feature selection and nomogram parameter determination based on equation 2; (**c**) simultaneous feature selection and nomogram parameter determination based on equation 2; (**d**) simultaneous feature selection and nomogram parameter determination based on equation 2; (**e**) perform feature selection based on equation 2, followed by nomogram parameter determination using equation 1.

After that, the Cox (Two-Stage) model chose a train cohort to do the univariate and multivariate cox regression analysis as the feature selection stage and the final one for the nomogram construction stage. Lasso-Cox, Ridge-Cox, and Elastic Net-Cox are all one-stage panelized cox proportional hazards models, where feature selection and the final nomogram model construction are done simultaneously during the building process. During the building of the one-stage model, the optimal parameter estimation was achieved using five-fold cross-validation. Cox With Lasso model is also a two-stage Cox model, where the features picked by the Lasso-Cox model are incorporated into the Cox model and the nomogram is created at the same time (Fig. 2). Time-dependent receiver operating characteristic (ROC) was implemented as an estimate of the cumulative/dynamic area under the curve (AUC) for a given collection of time points. Six-time points are included: 6 months, 1 year, 3 years, 5 years, 7 years, and 10 years. At each time point, the AUC rankings for the first and top three are determined.

### Institutional review board statement

All data are publicly available, and no IRB was required.

### Informed consent statement

Patient consent was waived due to this article using data from the SEER database, which is publicly available deidentified patient data from the National Cancer Institute (NCI), USA.

## Results

In the SEER database, 5463 GISTs patients were enlisted in this study. Whether in the OS or CSS tasks, the variables are arranged in increasing order of missing rates as follows: Tumor size, Marital status at diagnosis, Tumor location, AJCC Stage, Mitotic rate, and Tumor grade (Fig. S1-2). So, following this sorting order, the results produced after utilizing the MissCatBoosts algorithm for imputing missing data, conducting statistical analysis, modeling, and evaluation are as follows:

### Demographic traits

**Figure 3:**
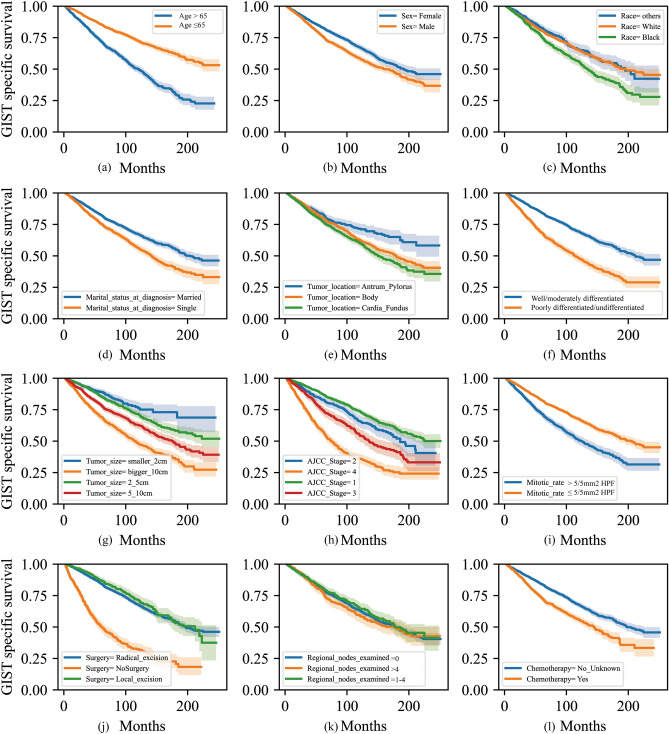
Kaplan–Meier curves stratified by patient characteristics in OS and the Log-rank tests for all subplots were p < 0.05: (**a**) Age, (**b**) Sex, (**c**) Race, (**d**) Marital status at diagnosis, (**e**) Tumor location, (**f**) Tumor grade, (**g**) Tumor size, (**h**) AJCC Stage, (**i**) Mitotic rate, (**j**) Surgery, (**k**) Regional nodes examined, (**l**) Chemotherapy.

All categorical variables for OS are depicted in Kaplan–Meier survival curves in Fig. 3. According to the Log-rank test, all factors exhibit p-values less than 0.05. Notably, age has been categorized using a threshold of 65, as displayed in Fig. S3. It is interesting that Tumor Grade and Mitotic Rate demonstrate a high degree of similarity, which may have an impact on feature selection and modeling. Similar patterns are also detected in the Kaplan–Meier curves for CSS, as illustrated in Fig. S4. However, it is crucial to highlight that the Log-Rank test is often viewed as a very forgiving non-parametric test with poorer sensitivity. Typically, stringent multiple comparison corrections are not required when conducting multiple comparisons with this test. This may improve the likelihood of preserving the null hypothesis. Therefore, both Kaplan–Meier survival curves and the Log-Rank test are applied exclusively for analyzing trends and making early comparisons in survival data.

Demographics and trends are reported in Table 1. The median age and interquartile range for age were 64 [54, 73]. When grouped by gender, 48.5% (n = 2650) were female, and 51.5% (n = 2813) were male, maintaining an overall gender ratio close to 1:1. Regarding ethnicity, 62.4% (n = 3409) were White, 22.6% (n = 1234) were Black, and 15.0% (n = 820) were classed as Others (including American Indians/Alaska Natives and Asian/Pacific Islanders).

**Table 1:** Demographics and train-test cohort split of 5463 patients with gastric GISTs from the Surveillance, Epidemiology, and End Results (SEER) database for the years 2000 to 2020.

Variable	Whole population	Train cohort	Test cohort	p-value*
Total patients	5463 (100%)	3824 (70%)	1639 (30%)	
Age_at_diagnosis	64.0 [54, 73]	63 [54, 73]	64 [55, 72]	0.554
Sex				
Male	2650 (48.5%)	1843(48.2%)	807(49.2%)	0.499
Female	2813 (51.5%)	1981(51.8%)	832(50.8%)	0.499
Race				
White	3409 (62.4%)	2395(62.6%)	1014(61.9%)	0.615
Black	1234 (22.6%)	853(22.3%)	381(23.2%)	0.468
Others	820 (15.0%)	576(15.1%)	244(14.9%)	0.900
Marital_status_at_diagnosis				
Married	3321 (60.8%)	2320 (60.7%)	1011 (61.1%)	0.802
Single	2142 (39.2%)	1504 (39.3%)	638 (38.9%)	0.802
Tumor_location				
Antrum or Pylorus	622 (11.4%)	435 (11.4%)	187 (11.4%)	1.000
Body	2971 (54.4%)	2058 (53.8%)	913 (55.7%)	0.210
Cardia or Fundus	1870 (34.2%)	1331 (34.8%)	539 (32.9%)	0.180
Tumor_grade				
Well/moderately differentiated	3939 (72.1%)	2757 (72.1%)	1182 (72.1%)	1.000
Poorly differentiated/undifferentiated	1524 (27.9%)	1067 (27.9%)	457 (27.9%)	1.000
Tumor_size				
≤ 2 cm	697 (12.8%)	491 (12.8%)	206 (12.6%)	0.817
2–5 cm	1782 (32.6%)	1247 (32.6%)	535 (32.6%)	1.000
5–10 cm	1677 (30.7%)	1181 (30.9%)	496 (30.3%)	0.671
≥ 10 cm	1307 (23.9%)	905 (23.7%)	402 (24.4%)	0.516
AJCC stage				
I	3031 (55.5%)	2132 (55.8%)	899 (54.9%)	0.558
II	734 (13.4%)	501 (13.1%)	233 (14.2%)	0.287
III	676 (12.4%)	487 (12.7%)	189 (11.5%)	0.233
IV	1022 (18.7%)	704 (18.4%)	318 (19.4%)	0.410
Mitotic_rate				
≤ 5/5 mm^2^ HPF	4081 (74.7%)	2853 (74.6%)	1228 (74.9%)	0.832
> 5/5 mm^2^ HPF	1382 (25.3%)	971 (25.4%)	411 (25.1%)	0.832
Surgery				
Local excision	608 (11.1%)	409 (10.7%)	199 (12.1%)	0.131
Radical_excision	4010 (73.4%)	2836 (74.2%)	1174 (71.6%)	0.056
No_Surgery	845 (15.5%)	579 (15.1%)	266 (16.2%)	0.328
Regional_nodes_examined				
0	4177 (76.5%)	2916 (76.3%)	1261 (76.9%)	0.610
1–4	675 (12.4%)	477 (12.5%)	198 (12.1%)	0.719
> 4	611 (11.2%)	431 (11.3%)	180 (11.0%)	0.792
Chemotherapy				
Yes	2175 (39.8%)	1497 (39.1%)	678 (41.4%)	0.132
No/Unknown	3288 (60.2%)	2327 (60.9%)	961 (58.6%)	0.132

*Kolmogorov–Smirnov test for numeric variables and chi-squared test for numeric variables.

To ease subsequent model creation and successful model assessment, the 5463 patients were randomly separated into train and test cohorts in a 7:3 ratio, assuring consistent proportions for each category with no statistical differences. Age, handled as a numerical variable, had a median and interquartile range of 63 [54, 73] in the Train cohort and 64 [55, 72] in the Test cohort, with a matching Kolmogorov–Smirnov test p-value of 0.554. All other variables were categorical, and the chi-squared test was applied. For instance, in the Tumor Size category, the proportions in the Whole Population were as follows: ≤ 2 cm—12.8% (n = 697), in the Train cohort—12.8% (n = 491), and in the Test cohort—12.6% (n = 206), with a p-value of 0.817. The proportions for the 2–5 cm category were: Whole Population—32.6% (n = 1782), Train cohort—32.6% (n = 1782), and Test cohort—32.6% (n = 1247), with a p-value of 1.000. Similarly, the 5–10 cm and ≥ 10 cm categories revealed similar proportions and no significant variations in the train and test cohorts. The demographic parameters and train-test cohort split for OS patients followed the same process, as stated in Table S2.

It is crucial to highlight that even when the prerequisites of preserving consistency with the general distribution and assuring no statistical disparities in the train and test cohorts divisions are met, there may still be intrinsic randomness affecting subsequent modeling and evaluation. Therefore, this study completed 50 rounds of data splitting for both OS and CSS patient groups, with each iteration satisfying the aforementioned consistency and no statistical difference requirements.

### Construction of nomogram models

The construction of the Nomogram model primarily involves two-stage which are feature selection and model building conducted separately, and a single-stage approach where feature selection and the final model construction occur simultaneously. In the two-stage Cox Nomogram, we first perform both univariate and multivariate feature selection (Tables 2, S3). In the univariate selection stage, all categories of 'Regional nodes examined' exhibited no statistically significant differences in OS. In the multivariate selection stage, elements showing statistical significance in the univariate stage are included.

**Table 2:** Univariate and multivariate Cox regression analyses of OS from the constructed nomogram.

Variable	Univariate analysis			Multivariate analysis		
	HR*	95% CI*	p value	HR*	95% CI*	p value
Age_at_diagnosis	1.04	1.04–1.05	< 0.005	1.04	1.04–1.05	< 0.005
Sex						
Female	Reference	–	–	–	–	–
Male	1.26	1.12–1.43	< 0.005	1.40	1.23–1.60	< 0.005
Race						
White	Reference	–	–	–	–	–
Black	1.40	1.22–1.61	< 0.005	1.43	1.24–1.65	< 0.005
Others	0.91	0.76–1.10	0.33	-	-	-
Marital_status_at_diagnosis						
Married	Reference	–	–	–	–	–
Single	1.46	1.29–1.65	< 0.005	1.33	1.17–1.52	< 0.005
Tumor_location						
Antrum or Pylorus	Reference	–	–	–	–	–
Body	1.28	1.03–1.58	0.02	1.11	0.90–1.39	0.33
Cardia or Fundus	1.57	1.26–1.95	< 0.005	1.17	0.93–1.46	0.17
Tumor_grade						
Well/moderately differentiated	Reference	–	–	–	–	–
Poorly differentiated/undifferentiated	2.09	1.84–2.36	< 0.005	1.34	1.13–1.59	< 0.005
Tumor_size						
≤ 2 cm	Reference	–	–	–	–	–
2–5 cm	1.46	1.10–1.93	0.01	1.28	0.96–1.70	0.09
5–10 cm	2.04	1.55–2.68	< 0.005	1.68	1.26–2.24	< 0.005
≥ 10 cm	3.11	2.37–4.09	< 0.005	1.97	1.43–2.70	< 0.005
AJCC* stage						
I	Reference	–	–	–	–	–
II	1.34	1.10–1.64	< 0.005	1.17	0.90–1.51	0.24
III	1.86	1.55–2.23	< 0.005	1.40	1.03–1.90	0.03
IV	3.47	3.00–4.01	< 0.005	1.81	1.42–2.32	< 0.005
Mitotic_rate						
≤ 5/5 mm^2^ HPF*	Reference	–	–	–	–	–
> 5/5 mm^2^ HPF*	1.69	1.49–1.92	< 0.005	0.89	0.71–1.11	0.31
Surgery						
Local excision	Reference	–	–	–	–	–
Radical_excision	1.15	0.93–1.43	0.20	–	–	–
No_Surgery	4.18	3.31–5.28	< 0.005	2.24	1.87–2.69	< 0.005
Regional_nodes_examined						
0	Reference	–	–	–	–	–
1–4	0.93	0.77–1.11	0.43	–	–	–
> 4	1.09	0.90–1.30	0.38	–	–	–
Chemotherapy						
Yes	1.57	1.39–1.77	< 0.005	0.95	0.82–1.10	0.52
No/Unknown	Reference	–	–	–	–	–

*HR: hazard ratio; CI: confidence interval, AJCC stage: American Joint Committee on Cancer stage; HPF: high-power microscopic fields.

In the analysis of the OS, a total of 10 elements were included. The hazard ratios (HR) [95% confidence intervals] and corresponding p-values for each element are presented as follows: Age at diagnosis with an HR of 1.04 [1.04–1.05], p < 0.005; Male with an HR of 1.40 [1.23–1.60], p < 0.005; Black with an HR of 1.43 [1.24–1.65], p < 0.005; Single with an HR of 1.33 [1.17–1.52], p < 0.005; Poorly differentiated/undifferentiated with an HR of 1.34 [1.13–1.59], p < 0.005; 5-10 cm with an HR of 1.68 [1.26–2.24], p < 0.005; ≥ 10cm with an HR of 1.97 [1.43–2.70], p < 0.005; AJCC_III with an HR of 1.40 [1.03–1.90], p = 0.03; AJCC_IV with an HR of 1.81 [1.42–2.32], p < 0.005; No_Surgery with an HR of 2.24 [1.87–2.69], p < 0.005 (Table 2). In the context of CSS, a total of 11 elements were included in the analysis. The results of this analysis are presented in Table S3, which includes the corresponding hazard ratios (HR) along with their respective 95% confidence intervals (CI) and p-values. The HR and CI for each element are as follows: Age_at_diagnosis HR = 1.03 [1.02–1.05], p < 0.005; Male HR = 1.60 [1.17–2.18], p < 0.005; Black HR = 1.70 [1.22–2.36], p < 0.005; Single HR = 1.41 [1.03–1.95], p = 0.03; Poorly differentiated/undifferentiated HR = 1.56 [1.09–2.23], p = 0.02; 5–10 cm HR = 2.47 [1.58–3.86], p < 0.005; ≥ 10 cm HR = 2.84 [1.72–4.69], p < 0.005; AJCC_III HR = 2.45 [1.26–4.77], p = 0.01; AJCC_IV HR = 2.16 [1.22–3.83], p = 0.01; No_Surgery HR = 4.92 [2.27–10.66], p < 0.005; Regional_nodes_examined > 4 HR = 1.59 [1.06–2.38], p = 0.02 (Table S3).

The Lasso-Cox model of OS identified 17 non-zero coefficient features in the one-stage Cox Nomogram. The optimal lambda value, determined through five-fold cross-validation, was found to be 0.002759628275668862 (Fig. S5). The Ridge-Cox model of OS identified 26 non-zero coefficient features, and the optimal lambda value determined by five-fold cross-validation was found to be 0.014727306097177213. The Elastic Net-Cox model of OS identified 21 features with non-zero coefficients. The optimal lambda value, determined using five-fold cross-validation, was found to be 0.00634580382929533 with a gamma value of 0.5 (refer to Fig. S6). The Lasso-Cox model of CSS identified 21 non-zero coefficient features, with a best lambda value of 0.000723002378351044 determined using five-fold cross-validation. The Ridge-Cox model of CSS identified 31 non-zero coefficient features, and the optimal lambda value, determined using five-fold cross-validation, was found to be 0.0008913511247589448. The Elastic Net-Cox model of CSS identified 26 features with non-zero coefficients. The optimal lambda value, determined using five-fold cross-validation (Fig. S7), was found to be 0.0006711759532059377 with a gamma value of 0.5 (Fig. S7).

We created an additional two-stage Cox Nomogram model, specifically the Cox model with Lasso, utilizing either the 17 non-zero coefficient features from the Lasso-Cox model of OS or the 21 non-zero coefficient features from the Lasso-Cox model of CSS. The models underwent retraining using the train cohort as the basis for developing the OS and CSS Nomogram models (Fig. 2).

### Time-dependent evaluation

The sensitivity and specificity of diagnostic tests are influenced by the dynamic and evolving nature of a patient's disease status. As a result, the ROC curve is extended to incorporate continuous outcomes.

**Figure 4:**
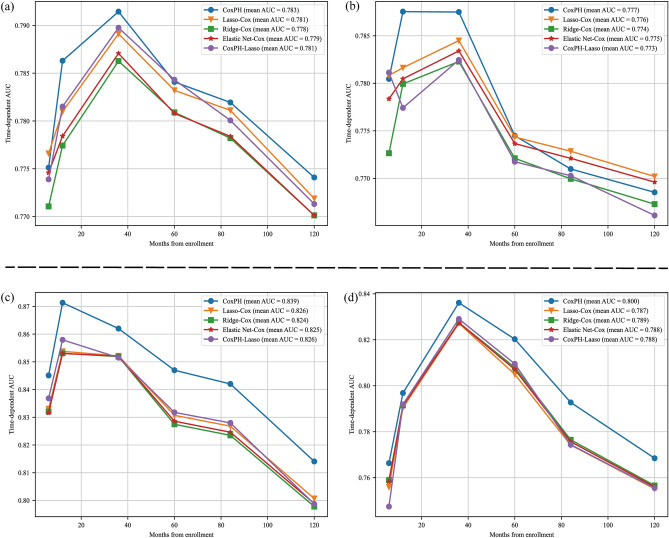
The receiver operating characteristic curves of the nomograms for half-, 1-, 3-, 5-, 7-, 10-year in the test cohort. (**a**, **b**) Different random partitions in OS, which ensure there are no statistically significant differences in the distributions between the train and test cohorts; (**c**, **d**) different random partitions in CSS, which ensure there are no statistically significant differences in the distributions between the train and test cohorts.

Table 3 provides a detailed analysis of five OS Cox nomograms across six distinct time intervals. Among the 50 experiments conducted, it was found that the Cox (Two-Stage) model demonstrated superior performance in terms of mean AUC [25–75%], top1 ratio, and top3 ratio across various time intervals. Specifically, at the Half a Year mark, the AUC was 0.809 [0.781–0.836], the Top1 ratio was 35 out of 50, and the Top3 ratio was 50 out of 50. Similarly, at the One Year mark, the AUC was 0.793 [0.774–0.813], the Top1 ratio was 46 out of 50, and the Top3 ratio was 50 out of 50. The trend continued with the Three Years mark, where the AUC was 0.776 [0.760–0.787], the Top1 ratio was 34 out of 50, and the Top3 ratio was 45 out of 50. Moving on to the Five Years mark, the AUC was 0.778 [0.764–0.789], the Top1 ratio was 33 out of 50, and the Top3 ratio was 42 out of 50. At the Seven Years mark, the AUC was 0.771 [0.761–0.782], the Top1 ratio was 30 out of 50, and the Top3 ratio was 47 out of 50. Finally, at the Ten Years mark, the AUC was 0.775 [0.766–0.783], the Top1 ratio was 26 out of 50, and the Top3 ratio was 38 out of 50. Figure 4a, b presents a graphical depiction of the performance exhibited by the five models on the test set across various random partitions. It is worth mentioning that the Cox (Two-Stage) model utilizes a smaller set of features, specifically 10, in comparison to comparable single-stage models such as Lasso-Cox with 17 features, Ridge-Cox with 26 features, and Elastic Net-Cox with 21 features. The Cox With Lasso model, which follows a two-stage approach, employs the 17 features extracted from the single-stage Lasso-Cox model to train the Cox model. This two-stage model demonstrates a performance that is only surpassed by the Cox (Two-Stage) model.

**Table 3:** Comprehensive comparative summary table of 5 OS cox nomograms at six-time points.

Models	AUC[25%-75%]	Top1	Top3	AUC[25%-75%]	Top1	Top3
Near-term	Half a Year			One Year		
Cox(Two-Stage)	**0.809 **[0.781–0.836]	**35/50**	**50/50**	**0.793 **[0.774–0.813]	**46/50**	**50/50**
Lasso-Cox	0.806 [0.777–0.834]	2/50	48/50	0.788 [0.768–0.805]	0/50	48/50
Ridge-Cox	0.802 [0.771–0.832]	0/50	0/50	0.786 [0.766–0.804]	0/50	4/50
Elastic Net-Cox	0.805 [0.776–0.833]	0/50	15/50	0.787 [0.768–0.805]	0/50	19/50
Cox With Lasso	0.807 [0.778–0.837]	**13/50**	37/50	0.788 [0.770–0.805]	**4/50**	29/50

Mid-term	Three Years			Five Years		
Cox(Two-Stage)	**0.776 **[0.760–0.787]	**34/50**	43/50	**0.778 **[0.764–0.789]	**33/50**	**42/50**
Lasso-Cox	0.774 [0.759–0.785]	2/50	**45/50**	0.777 [0.762–0.786]	4/50	39/50
Ridge-Cox	0.773 [0.758–0.784]	2/50	10/50	0.776 [0.761–0.785]	1/50	23/50
Elastic Net-Cox	0.774 [0.759–0.784]	2/50	18/50	0.776 [0.761–0.785]	1/50	13/50
Cox With Lasso	0.775 [0.760–0.785]	**10/50**	34/50	0.777 [0.763–0.788]	**11/50**	33/50

Long-term	Seven Years			Ten Years		
Cox(Two-Stage)	**0.771 **[0.761–0.782]	**30/50**	**47/50**	**0.775 **[0.766–0.783]	**26/50**	**38/50**
Lasso-Cox	0.769 [0.760–0.779]	5/50	39/50	0.774 [0.763–0.780]	2/50	35/50
Ridge-Cox	0.769 [0.759–0.780]	6/50	20/50	0.774 [0.763–0.782]	5/50	27/50
Elastic Net-Cox	0.769 [0.759–0.779]	2/50	18/50	0.774 [0.763–0.781]	**13/50**	33/50
Cox With Lasso	0.769 [0.758–0.780]	**7/50**	26/50	0.773 [0.763–0.781]	4/50	17/50

Significant values are in [bold].

Table S4 provides a complete comparison of five CSS Cox nomograms at six distinct time intervals. The Cox (Two-Stage) model exhibited superior performance across multiple time intervals in a total of 50 experiments. Specifically, it demonstrated the highest mean AUC [25–75%], top1 ratio, and top3 ratio at various time intervals. For instance, at the one-year mark, the Cox (Two-Stage) model achieved an AUC of 0.831 [0.797–0.861], a top1 ratio of 21 out of 50, and a top3 ratio of 26 out of 50. Similarly, at the three-year mark, it attained an AUC of 0.837 [0.823–0.851], a top1 ratio of 45 out of 50, and a top3 ratio of 49 out of 50. The model's performance remained consistently high at the five-year, seven-year, and ten-year intervals, with AUC values of 0.836 [0.827–0.846], 0.824 [0.810–0.841], and 0.814 [0.798–0.828], respectively. Additionally, the top1 and top3 ratios remained consistently high at these intervals, with the model achieving ratios of 50 out of 50 and 50 out of 50, respectively. Figure 4c, d visually illustrate the performance of the five models on the test dataset across various random partitions. It is worth mentioning that the Cox (Two-Stage) model has a total of 11 features, which is notably lower compared to the other single-stage models, namely Lasso-Cox with 21 features, Ridge-Cox with 31 features, and Elastic Net-Cox with 26 features. The Cox With Lasso model, which is a two-stage model, utilizes 21 features from the single-stage Lasso-Cox model for training the Cox model. This approach ensures that the performance of Cox With Lasso is at least as good as that of Lasso-Cox in general.

### Best model presentation

**Figure 5:**
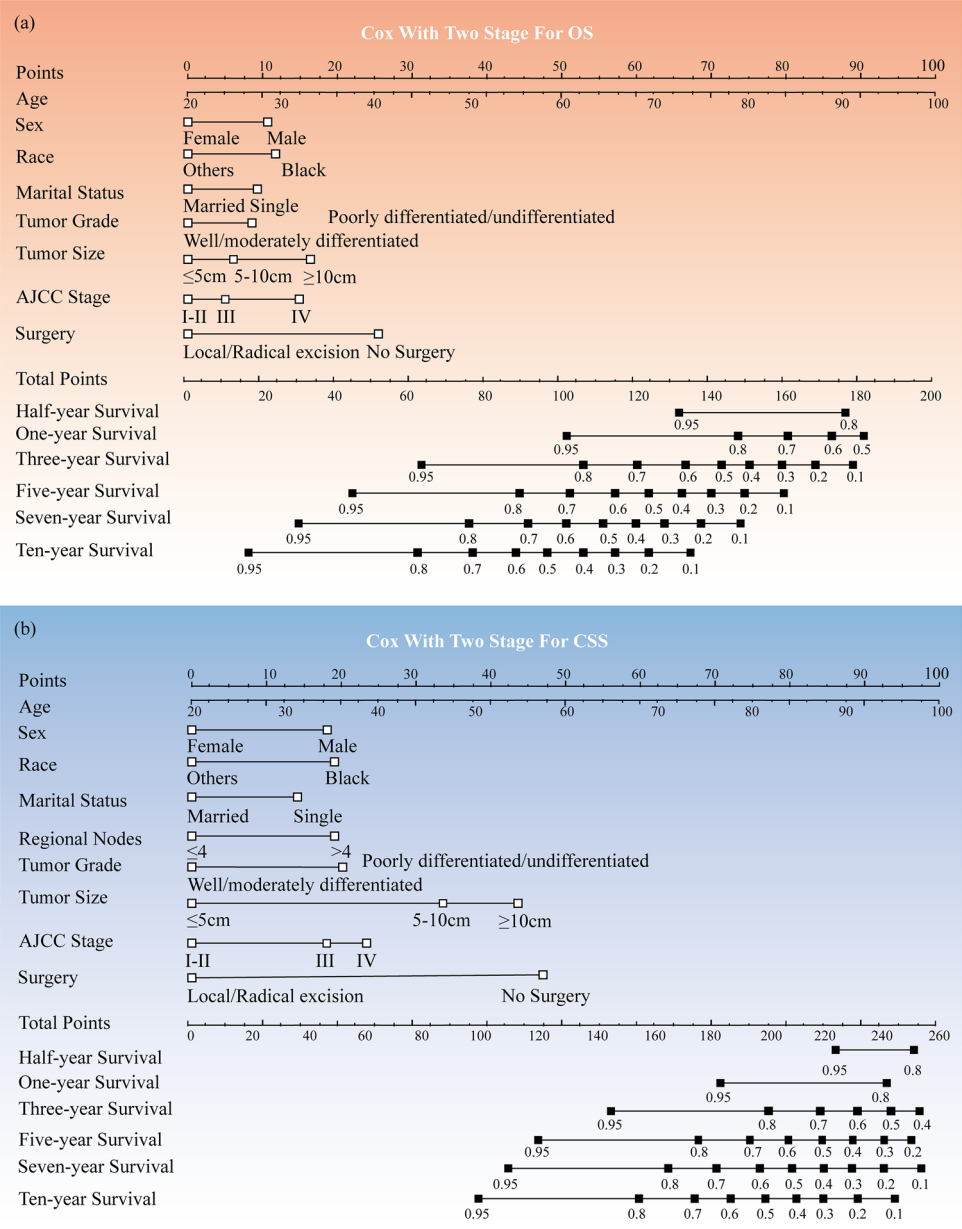
Nomogram for predicting half-year, one-year, three-year, five-year, seven-year, and ten-year in patients with gastric GISTs: (**a**) Cox with two-stage for OS nomogram; (**b**) Cox with two-stage for CSS nomogram.

Based on the comparison data shown above, it can be observed that the Cox (Two-Stage) model demonstrates a notable degree of simplicity and effectiveness in predicting both OS and CSS. Furthermore, the Cox With Lasso approach presents an additional and potentially advantageous alternative option. The clinical validity of the Cox with Two-Stage nomograms for OS and CSS has been documented (refer to Fig. 5). In addition, Fig. S8 presents the prospective models derived from the Penalized Cox Model, specifically the Cox with LASSO Features nomograms for OS and CSS.

## Discussion

Despite the high probability of recurrence subsequent to surgery, surgical excision remains the predominant therapeutic approach for primary GISTs^3^. The extended viability of GISTs sometimes leads to patient mortality resulting from factors unrelated to GISTs. Consequently, prognostic estimates of OS may not adequately reflect the long-term survival rates of GISTs. Hence, this study aimed to assess and compare the overall performance of five Cox nomogram models for OS and CSS at six distinct time intervals. The objective was to determine the most straightforward and reliable nomogram model for predicting survival in patients with gastric GISTs. In order to mitigate the potential confounding effects caused by comparing different nomograms between the train and test cohorts, we implemented cross-validation during the model development process. We performed 50 distinct train and test cohort splits for both OS and CSS. This approach ensured that all variables exhibited no statistically significant differences within each split, as indicated in Tables 1 and S2. Our study incorporated a comprehensive and extensive sample size, derived from the newest SEER database, which included 17 registries and encompassed a varied range of ethnic communities. This was particularly significant due to the infrequency of gastric GISTs.

The nomogram is a statistical instrument that has resemblance to clinical rating scales, while it distinguishes itself from such scales in terms of its derivation. Clinical nomograms are commonly developed from Cox regression or logistic regression models, allowing for the conversion of any Cox regression model into a clinical nomogram. The focus of this work is to five discrete nomograms that correlate to unique Cox regression models. Previous research has indicated that nomograms are increasingly being seen as prospective substitutes for the National Institute of Health (NIH) criteria, SEER staging, and TNM staging systems. These nomograms have the capacity to potentially establish a novel clinical standard^7,8,10,12^. However, the existing literature oversimplifies the treatment of missing data classified as 'unknown' in the development of nomograms. Several research^7,8,10,11^ consider the classification of 'unknown' as a distinct category. The validity of this assumption is frequently disregarded, as it is contingent upon the proportions of categories inside the 'unknown' group being similar to the overall proportions. However, this condition is rarely satisfied and poses difficulties in its verification. In the majority of instances, the introduction of randomness from 'unknown' sources poses obstacles to the repeatability and generalizability of models. This study was motivated by the presence of multiclass variables in SEER databases. In order to address this issue, the researchers drew inspiration from a Multiple Imputation technique^22^ and proposed a novel algorithm called MissCatBoosts. The methodology employed in this study involves the utilization of CatBoost^23^, a well-established technology renowned for its ability to effectively handle categorical variables, as the foundational learner. The program employs a methodology that estimates missing values by taking into account variables with different levels of missingness. It then combines these estimates using an ensemble learning approach. Validation in real-world data sets is crucial for algorithmic performance assessment. In this study, we introduced artificial missing values into a prominent medical dataset from the University of California, Irvine (UCI) repository. We then benchmarked our MissCatboosts algorithm against three other leading methods. The results indicate superior performance of MissCatboosts for both binary and multiclass variables, as detailed in Table S5.

Nomograms frequently involve the presence of multicategory variables, which can be either ordinal or interval in nature. The influence on results frequently exhibits variability as categories increase or decrease. Nevertheless, previous research on nomograms for prognosticating survival in patients with GISTs has treated multicategory factors, including primary site, grade, and mitotic rate, as if they were numerical variables. This methodology enforces a consistent impact on results across many categories. For instance, in the case of the primary site variable, the influence on outcomes remains consistent during the passage from the stomach to the small intestine, from the small intestine to the rectum, from the rectum to the colon, and from the colon to additional sites. In a similar vein, the uniform treatment of the impact on outcomes is observed for the grade variable during the transitions from grade I to II, grade II to III, grade III to IV, and grade IV to 'unknown.' However, it is important to note that the impact on survival outcomes for the mitotic rate variable is unlikely to be consistent when transitioning from < 5 to 5–10, 5–10 to > 10, and > 10 to 'unknown' in clinical decision-making^10^. Hence, the multicategory variables in this study, such as Tumor Size, consistently demonstrate the true influence of various categories on survival outcomes in both OS and CSS nomograms. When the size of a tumor transitions from being less than or equal to 5 cm to being within the range of 5-10 cm, the effect on OS outcomes is more significant compared to CSS outcomes. In relation to the AJCC Stage variable, the influence on CSS outcomes is more pronounced compared to OS while progressing from AJCC Stage I-II to III. Conversely, the scenario is flipped when migrating from AJCC Stage III to IV.

The prognostic significance of the Mitotic rate has been widely acknowledged in recent clinical guidelines, such as those provided by the National Comprehensive Cancer Network (NCCN), French Intergroup Clinical Practice guidelines, the European Society for Medical Oncology (ESMO), and the European Reference Network on Rare Adult Cancers (EURACAN)^24–26^. Nevertheless, previous research has indicated that the Mitotic rate did not display any significant variations in survival outcomes when undergoing multifactor feature selection. The observed disparity is incongruous with the clinical guidelines indicated earlier^7,8,10,11^. The veracity is found within the survival Cox nomograms, which incorporate the independent clinical factor Mitotic rate and the comprehensive index AJCC stage as simultaneous variables in the nomogram. There is a certain degree of association between the AJCC stage and the Mitotic rate, as indicated by Fig. S9. In the context of multifactor feature selection, it is seen that both AJCC stage and the Mitotic rate exert a significant influence on survival outcomes. Consequently, these two factors are prioritized and included in the final selection process. It is noteworthy that three single-stage Cox nomogram models for CSS, including Lasso-Cox, Ridge-Cox, and Elastic Net-Cox, have retained both Mitotic rate and AJCC stage variables, indicating a possible advantage. Nevertheless, it is important to note that all three models have a shared limitation, which is the application of uniform penalties to all variables inside the model (Fig. 2)^27,28^. As a result of this constraint, the one-stage Cox nomogram maintains the inclusion of Mitotic rate and AJCC stage for CSS, hence exhibiting a more pronounced distinction in comparison to both the two-stage Cox nomogram and OS (refer to Tables 3, S4, and Fig. 4).

In this study, the performance of the two-stage Cox model with Lasso feature selection, also known as 'Cox With Lasso', was shown to be comparable to that of the classic two-stage Cox model, 'Cox (Two-Stage).' The observed difference in performance between the two models was rather minor. The construction of Cox nomograms (Fig. S8) continues to be a clinically promising strategy. Despite the study's limited inclusion of clinical variables, it remains an effective approach for feature selection, particularly in clinical scenarios involving high-dimensional characteristics like genomes^29,30^, proteomics^31,32^, and radiomics^14–17^.

This study is subject to many constraints. Firstly, it is important to note that this study is retrospective in nature, despite being derived from the biggest multicenter database for gastric GISTs that is currently accessible. This retrospective design may potentially bring inherent biases that should be taken into consideration when interpreting the findings. In order to corroborate the results, it is necessary to obtain a comprehensive dataset from multiple clinical centers in a prospective manner. Furthermore, it is important to note that the data utilized in this investigation did not encompass information predating the year 2000. Moreover, the SEER data employed in this analysis did not offer insights into the administration of imatinib mesylate or other targeted therapy to patients with gastric GISTs. In addition, it should be noted that prognosis can also be influenced by other factors such as tumor rupture, bleeding, and certain gene mutation types. However, it is important to mention that these factors were not incorporated into the nomograms due to their unavailability in the database. Research that leverages the SEER database provides valuable clinical insights; nevertheless, it is imperative to acknowledge its intrinsic limitations. While this investigation has employed methodologies to mitigate particular concerns, challenges such as potential biases and data incompleteness persist as underlying issues. Furthermore, future explorations could benefit from more nuanced approaches, including stratified analyses of variables such as marital status.

In addition to the analytical validation of our nomogram models, we have made these predictive tools accessible to a global audience by deploying them online. The nomogram models for prognostic prediction in gastric gastrointestinal stromal tumors (GISTs) are now available through a user-friendly interface at the following URL: https://gists-llyong.streamlit.app. This web application allows clinicians and researchers worldwide to input patient-specific data and receive immediate prognostic predictions, thereby facilitating informed decision-making in the management of gastric GISTs.

## Conclusions

When developing nomograms to predict OS and CSS in patients with gastric GISTs, it is important to appropriately handle multicategory variables and minimize the randomness caused by dividing the data into training and testing cohorts. In this regard, a two-stage Cox nomogram demonstrates superior performance compared to a single-stage penalty-based Cox nomogram. Nevertheless, the utilization of the two-stage Cox nomogram, which incorporates LASSO feature selection, continues to exhibit substantial promise. This finding serves as a catalyst for additional investigation into the development of multimodal survival prediction models for gastric GISTs.

### Supplementary Information

Supplementary Information.

## Data Availability

All data are publicly available.
