# Supplementary material for: Evaluating nomogram models for predicting survival outcomes in gastric gastrointestinal stromal tumors with SEER database analysis
Source: Sci Rep. 2024 May 20;14:11494. doi: 10.1038/s41598-024-62353-z (PMC11106332; doi:10.1038/s41598-024-62353-z)
Supplement: Supplementary file 1 — Supplementary Information. [file 41598_2024_62353_MOESM1_ESM.docx]

Supplemental material:

**Figure S1.** Missing value distribution diagram of features involved in OS modeling prediction, with missing proportions increasing sequentially in the order of 'Tumor size, Marital status at diagnosis, Tumor location, AJCC Stage, Mitotic rate, Tumor grade'"

**Figure S2.** Missing value distribution diagram of features involved in CSS prediction, with missing proportions increasing sequentially in the order of 'Tumor size, Marital status at diagnosis, Tumor location, AJCC Stage, Mitotic rate, Tumor grade'.

**Figure S3.** Statistical illustration of numerical features and missingness correlation of categorical variables. The age distribution of (**a**) OS and (**b**) CSS shows similarity and can be divided based on guidelines with a cutoff of 65 for calculating K-M curves. In both (**c**) OS and (**d**) CSS, missing values exhibit a clear positive correlation tendency due to their association with mitotic rate, tumor grade, and AJCC stage determinations.

**Figure S4.** Kaplan-Meier curves stratified by patient characteristics in CSS and the Log-rank tests for all subplots were p<0.05: (**a**) Age, (**b**) Sex, (**c**) Race, (**d**) Marital status at diagnosis, (**e**) Tumor location, (**f**) Tumor grade, (**g**) Tumor size, (**h**) AJCC Stage, (**i**) Mitotic rate, (**j**) Surgery, (**k**) Regional nodes examined, (**l**) Chemotherapy.

**Figure S5.** Lasso-Cox model feature selection illustration of OS. (**a**) 17 non-zero coefficients of Lasso-Cox model; (**b**) represents the Concordance Index values for different lambda values under five-fold cross-validation and the best lambda is 0.002759628275668862; (**c**) represents the coefficients of all features for different lambda values under five-fold cross-validation.

**Figure S6.** Ridge-Cox and Elastic Net-Cox model feature selection illustration of OS. (a) 26 non-zero coefficients of Ridge-Cox model; (b) represents the Concordance Index values for different lambda values under five-fold cross-validation and the best lambda is 0.014727306097177213; (c) represents the coefficients of all features for different lambda values under five-fold cross-validation; (d) 21 non-zero coefficients of Elastic Net-Cox model; (e) represents the Concordance Index values for different lambda values under five-fold cross-validation and the best lambda is 0.00634580382929533; (f) represents the coefficients of all features for different lambda values under five-fold cross-validation.

**Figure S7.** Lasso-Cox, Ridge-Cox and Elastic Net-Cox model feature selection illustration of CSS. (a) 21 non-zero coefficients of Lasso-Cox model; (b) represents the Concordance Index values for different lambda values under five-fold cross-validation and the best lambda is 0.000723002378351044; (c) represents the coefficients of all features for different lambda values under five-fold cross-validation; (d) 31 non-zero coefficients of Ridge-Cox model; (e) represents the Concordance Index values for different lambda values under five-fold cross-validation and the best lambda is 0.0008913511247589448; (f) represents the coefficients of all features for different lambda values under five-fold cross-validation; (g) 26 non-zero coefficients of Elastic Net-Cox model; (h) represents the Concordance Index values for different lambda values under five-fold cross-validation and the best lambda is 0.0006711759532059377; (i) represents the coefficients of all features for different lambda values under five-fold cross-validation.

**Figure S8.** Nomogram for predicting half-year, one-year, three-year, five-year, seven-year and ten-year in patients with gastric GISTs: (**a**) Cox with LASSO Features for OS nomogram; (**b**) Cox with LASSO Features for CSS nomogram.

**Figure S9.** Correlation Heatmap of Categorical Variables. (a) In OS, different categories of AJCC stage and Mitotic rate show a clear correlation; (b) In CSS, different categories of AJCC stage and Mitotic rate also exhibit a significant correlation.

**Table S1. The pseudo code table of MissCatBoosts**

Algorithm : MissCatBoosts Flow.

Make an initial guess for all missing categorical/numeric values (e.g. mean, mode)

$k \leftarrow$vector of column indices in $\boldsymbol{X}$, sorted in ascending order of % missing

Construction of CatBoost models with $m$ sets of distinct hyperparameters

while not $\gamma$ do:

$\boldsymbol{X}_{\boldsymbol{old}}^{\boldsymbol{imp}} \leftarrow$store previous imputed matrix

for $s$ in $k$ do:

fit a CatBoost model predicting the non-missing values of $\boldsymbol{X}_{\boldsymbol{s}}:\boldsymbol{y}_{\boldsymbol{obs}}^{(\boldsymbol{s})}\sim\boldsymbol{x}_{\boldsymbol{obs}}^{(\boldsymbol{s})}$

use this to predict the missing values of $\boldsymbol{X}_{\boldsymbol{s}}$: predict $\boldsymbol{y}_{\boldsymbol{mis}}^{(\boldsymbol{s})}$ using $\boldsymbol{x}_{\boldsymbol{mis}}^{(\boldsymbol{s})}$

$\boldsymbol{X}_{\boldsymbol{new}}^{\boldsymbol{imp}} \leftarrow$update imputed matrix, using the predicted $\boldsymbol{y}_{\boldsymbol{mis}}^{(\boldsymbol{s})}$

end for

update $\gamma$

end while

Aggregation of $m$ matrices to obtain the final imputed matrix $\boldsymbol{X}_{\boldsymbol{new}}^{\boldsymbol{imp}}$

return $\boldsymbol{X}_{\boldsymbol{new}}^{\boldsymbol{imp}} \leftarrow$

***MissCatBoosts algorithm pseudocode Requires:**

$\boldsymbol{X}$ , An $n\times p$ matrix of predictors that requires imputation;

$\gamma$ , The stopping criterion;

$\boldsymbol{X}_{\boldsymbol{s}}$, An arbitrary variable, which contains missing values at entries $i_{mis}^{(s)}\subseteq\left\{ 1,2,\ldots,n \right\}$

$\boldsymbol{y}_{\boldsymbol{obs}}^{(\boldsymbol{s})}$, The non-missing values of variable $\boldsymbol{X}_{\boldsymbol{s}}$;

$\boldsymbol{y}_{\boldsymbol{miss}}^{(\boldsymbol{s})}$, The missing values of variable $\boldsymbol{X}_{\boldsymbol{s}}$;

$\boldsymbol{x}_{\boldsymbol{miss}}^{(\boldsymbol{s})}$, The variables other than $\boldsymbol{X}_{\boldsymbol{s}}$, with observations $i_{mis}^{(s)}$;

$\boldsymbol{x}_{\boldsymbol{obs}}^{(\boldsymbol{s})}$, The variables other than $\boldsymbol{X}_{\boldsymbol{s}}$, with observations ${\left\{ 1,2,\ldots,n \right\} \setminus i}_{mis}^{(s)}$;

**Table S2.** Demographics and train-test cohort split of 4,238 patients with gastric gastrointestinal stromal tumors from the Surveillance, Epidemiology, and End Results (SEER) database for the years 2000 to 2020.

| **Variable** | **Whole population** | **Train cohort** | **Test cohort** | **p-value*** |
| --- | --- | --- | --- | --- |
| Total patients | 4238(100%) | 2966(70%) | 1272(30%) |  |
| Age_at_diagnosis | 62.0[53,71] | 62[53,71] | 62[54,70] | 0. 792 |
| Sex |  |  |  |  |
| Male | 1990 (47.0%) | 1388(46.8%) | 602(47.3%) | 0.777 |
| Female | 2248 (53.0%) | 1578(53.2%) | 670(52.7%) | 0.777 |
| Race |  |  |  |  |
| White | 2651 (62.6%) | 1869(63.0%) | 782(61.5%) | 0.362 |
| Black | 920 (21.7%) | 636(21.4%) | 284(22.3%) | 0.549 |
| Others | 667 (15.7%) | 461(15.5%) | 206(16.2%) | 0.625 |
| Marital_status_at_diagnosis |  |  |  |  |
| Married | 2651 (62.6%) | 1856(62.6%) | 795(62.5%) | 0.990 |
| Single | 1587 (37.4%) | 1110(37.4%) | 477(37.5%) | 0.990 |
| Tumor_location |  |  |  |  |
| Antrum or Pylorus | 515 (12.2%) | 356(12.0%) | 159(12.5%) | 0.687 |
| Body | 2243 (52.9%) | 1577(53.2%) | 666(52.4%) | 0.652 |
| Cardia or Fundus | 1480 (34.9%) | 1033(34.8%) | 447(35.1%) | 0.872 |
| Tumor_grade |  |  |  |  |
| Well/moderately differentiated | 3332 (78.6%) | 2341(78.9%) | 991(77.9%) | 0.483 |
| Poorly differentiated/undifferentiated | 906 (21.4%) | 625(21.1%) | 281(22.1%) | 0.483 |
| Tumor_size |  |  |  |  |
| ≤2 cm | 618 (14.6%) | 430(14.5%) | 188(14.8%) | 0.848 |
| 2-5cm | 1467 (34.6%) | 1017(34.3%) | 450(35.4%) | 0.517 |
| 5-10cm | 1262 (29.8%) | 885(29.8%) | 377(29.6%) | 0.925 |
| ≥10cm | 891 (21.0%) | 634(21.4%) | 257(20.2%) | 0.414 |
| AJCC Stage |  |  |  |  |
| Ⅰ | 2583 (60.9%) | 1816(61.2%) | 767(60.3%) | 0.594 |
| Ⅱ | 567 (13.4%) | 385(13.0%) | 182(14.3%) | 0.265 |
| Ⅲ | 497 (11.7%) | 352(11.9%) | 145(11.4%) | 0.702 |
| Ⅳ | 591 (13.9%) | 413(13.9%) | 178(14.0%) | 0.991 |
| Mitotic_rate |  |  |  |  |
| ≤5/5mm^2^ HPF | 3342 (78.9%) | 2345(79.1%) | 997(78.4%) | 0.647 |
| >5/5mm^2^ HPF | 896 (21.1%) | 621(20.9%) | 275(21.6%) | 0.647 |
| Surgery |  |  |  |  |
| Local excision | 473 (11.2%) | 340(11.5%) | 133(10.5%) | 0.367 |
| Radical_excision | 3252 (76.7%) | 2272(76.6%) | 980(77.0%) | 0.785 |
| No_Surgery | 513 (12.1%) | 354(11.9%) | 159(12.5%) | 0.642 |
| Regional_nodes_examined |  |  |  |  |
| 0 | 3246 (76.6%) | 2270(76.5%) | 976(76.7%) | 0.922 |
| 1-4 | 521 (12.3%) | 361(12.2%) | 160(12.6%) | 0.750 |
| >4 | 471 (11.1%) | 335(11.3%) | 136(10.7%) | 0.604 |
| Chemotherapys |  |  |  |  |
| Yes | 1633 (38.5%) | 1148(38.7%) | 485(38.1%) | 0.750 |
| No/Unknown | 2605 (61.5%) | 1818(61.3%) | 787(61.9%) | 0.750 |

* Kolmogorov-Smirnov test for numeric variables and chi-squared test for numeric variables.

**Table S3.** Univariate and multivariate Cox regression analyses of CSS from the constructed nomogram.

| **Variable** | **Univariate analysis** | | | **Multivariate analysis** | | |
| --- | --- | --- | --- | --- | --- | --- |
|  | **HR*** | **95% CI*** | **P value** | **HR*** | **95% CI*** | **P value** |
| Age_at_diagnosis | 1.03 | 1.02-1.05 | <0.005 | 1.03 | 1.02-1.05 | <0.005 |
| Sex |  |  |  |  |  |  |
| Female | Reference | - | - | - | - | - |
| Male | 1.69 | 1.26-2.27 | <0.005 | 1.60 | 1.17-2.18 | <0.005 |
| Race |  |  |  |  |  |  |
| White | Reference | - | - | - | - | - |
| Black | 2.15 | 1.56-2.96 | <0.005 | 1.70 | 1.22-2.36 | <0.005 |
| Others | 1.17 | 0.76-1.81 | 0.47 | - | - | - |
| Marital_status_at_diagnosis |  |  |  |  |  |  |
| Married | Reference | - | - | - | - | - |
| Single | 1.50 | 1.12-2.01 | 0.01 | 1.41 | 1.03-1.95 | 0.03 |
| Tumor_location |  |  |  |  |  |  |
| Antrum or Pylorus | Reference | - | - | - | - | - |
| Body | 1.54 | 0.90-2.67 | 0.12 | - | - | - |
| Cardia or Fundus | 1.91 | 1.10-3.33 | 0.02 | 1.11 | 0.81-1.52 | 0.53 |
| Tumor_grade |  |  |  |  |  |  |
| Well/moderately differentiated | Reference | - | - | - | - | - |
| Poorly differentiated/undifferentiated | 3.44 | 2.57-4.60 | <0.005 | 1.56 | 1.09-2.23 | 0.02 |
| Tumor_size |  |  |  |  |  |  |
| ≤2 cm | Reference | - | - | - | - | - |
| 2-5 cm | 1.39 | 0.60-3.20 | 0.44 | - | - | - |
| 5-10 cm | 3.95 | 1.81-8.62 | <0.005 | 2.47 | 1.58-3.86 | <0.005 |
| ≥10 cm | 7.44 | 3.44-16.09 | <0.005 | 2.84 | 1.72-4.69 | <0.005 |
| AJCC* Stage |  |  |  |  |  |  |
| Ⅰ | Reference | - | - | - | - | - |
| Ⅱ | 2.61 | 1.64-4.16 | <0.005 | 1.73 | 0.94-3.18 | 0.08 |
| Ⅲ | 5.13 | 3.44-7.66 | <0.005 | 2.45 | 1.26-4.77 | 0.01 |
| Ⅳ | 6.51 | 4.45-9.54 | <0.005 | 2.16 | 1.22-3.83 | 0.01 |
| Mitotic_rate |  |  |  |  |  |  |
| ≤5/5mm^2^ HPF* | Reference | - | - | - | - | - |
| >5/5mm^2^ HPF* | 2.71 | 2.02-3.64 | <0.005 | 1.06 | 0.67-1.68 | 0.81 |
| Surgery |  |  |  |  |  |  |
| Local excision | Reference | - | - | - | - | - |
| Radical_excision | 2.26 | 1.15-4.44 | 0.02 | 1.43 | 0.72-2.85 | 0.31 |
| No_Surgery | 2.22 | 4.53-18.65 | <0.005 | 4.92 | 2.27-10.66 | <0.005 |
| Regional_nodes_examined |  |  |  |  |  |  |
| 0 | Reference | - | - | - | - | - |
| 1-4 | 1.11 | 0.72-1.72 | 0.64 | - | - | - |
| >4 | 1.87 | 1.29-2.72 | <0.005 | 1.59 | 1.06-2.38 | 0.02 |
| Chemotherapy |  |  |  |  |  |  |
| Yes | 1.82 | 1.36-2.43 | <0.005 | 0.74 | 0.53-1.02 | 0.07 |
| No/Unknown | Reference | - | - | - | - | - |

* HR: hazard ratio; CI: confidence interval, AJCC stage: American Joint Committee on Cancer stage; HPF: high-power microscopic fields.

**Table S4.** Comprehensive comparative summary table of 5 CSS cox nomograms at 6 time points.

| **Models** | **AUC[25%-75%]** | **Top1** | **Top3** | **AUC[25%-75%]** | **Top1** | **Top3** |
| --- | --- | --- | --- | --- | --- | --- |
| **Near-term** | Half a Year | | | One Year | | |
| Cox(Two Stage) | 0.805[0.739-0.872] | 14/50 | 20/50 | **0.831[0.797-0.861]** | **21/50** | 26/50 |
| Lasso-Cox | 0.809[0.739-0.873] | 7/50 | 27/50 | 0.831[0.794-0.860] | 5/50 | 30/50 |
| Ridge-Cox | 0.811[0.736-0.870] | 9/50 | 37/50 | 0.831[0.793-0.858] | 8/50 | 30/50 |
| Elastic Net-Cox | 0.810[0.739-0.873] | 1/50 | **39/50** | 0.831[0.794-0.859] | 3/50 | **35/50** |
| Cox With Lasso | **0.810[0.743-0.869]** | **19/50** | 28/50 | 0.830[0.795-0.861] | **13/50** | 29/50 |
| **Mid-term** | Three Years | | | Five Years | | |
| Cox(Two Stage) | **0.837[0.823-0.851]** | **45/50** | **49/50** | **0.836[0.827-0.846]** | **50/50** | **50/50** |
| Lasso-Cox | 0.830[0.812-0.846] | 1/50 | 27/50 | 0.827[0.817-0.836] | 0/50 | 30/50 |
| Ridge-Cox | 0.830[0.812-0.846] | 0/50 | 24/50 | 0.827[0.818-0.836] | 0/50 | 20/50 |
| Elastic Net-Cox | 0.830[0.813-0.845] | 0/50 | 24/50 | 0.827[0.818-0.836] | 0/50 | 28/50 |
| Cox With Lasso | 0.830[0.812-0.845] | **4/50** | 26/50 | 0.826[0.817-0.835] | 0/50 | 22/50 |
| **Long-term** | Seven Years | | | Ten Years | | |
| Cox(Two Stage) | **0.824[0.810-0.841]** | **49/50** | **50/50** | **0.814[0.798-0.828]** | **48/50** | **48/50** |
| Lasso-Cox | 0.817[0.802-0.831] | 0/50 | 32/50 | 0.807[0.793-0.823] | 1/50 | 36/50 |
| Ridge-Cox | 0.816[0.801-0.831] | 1/50 | 24/50 | 0.806[0.794-0.821] | 0/50 | 19/50 |
| Elastic Net-Cox | 0.816[0.801-0.830] | 0/50 | 29/50 | 0.807[0.793-0.822] | 1/50 | 32/50 |
| Cox With Lasso | 0.815[0.800-0.830] | 0/50 | 15/50 | 0.805[0.793-0.820] | 0/50 | 15/50 |

**Table S5.** Benchmark comparison of models using Mean Absolute Error (MAE).

| **Models** | **Binary[1]** | | | **Multi-category[1]** | | | |
| --- | --- | --- | --- | --- | --- | --- | --- |
|  | **sex** | **restecg** | **exang** | **cp** | **slope** | **ca** | **thal** |
| MostFrequent | 0.78 | 0.50 | 0.27 | 0.88 | 1.48 | 0.80 | 1.37 |
| Mice[2] | 0.18 | 0.40 | 0.26 | 0.67 | 0.38 | 0.50 | 0.30 |
| MissForest[3] | 0.25 | 0.42 | 0.21 | 0.68 | 0.35 | 0.62 | 0.32 |
| MissCatboosts* | 0.16 | 0.40 | 0.22 | 0.68 | 0.27 | 0.62 | 0.28 |

* The imputation algorithm proposed in this paper.

For benchmarking comparisons of various missing value imputation algorithms, the following measures are implemented to uphold fairness and uniformity in our evaluations:

1. Random Seed: To maintain reproducibility, a single fixed random seed is designated and applied consistently across all models, ensuring that the stochastic processes involved in data shuffling and model initialization are equivalent.

2. Dataset: All models are trained and validated using an identical dataset, preserving the integrity of the performance comparisons.

3. Computational Resources: To negate the impact of differing computational capabilities on algorithmic performance, all models are executed on equivalent computing resources.

4. Evaluation Metrics: Performance metrics are standardized to provide an unbiased assessment of each model's efficacy, adhering to a common set of evaluation criteria.

References

1. Detrano, R., Janosi, A., Steinbrunn, W., et al. (1989). International application of a new probability algorithm for the diagnosis of coronary artery disease. Am. J. Cardiol. **64**, 304-310, doi: 10.1016/0002-9149(89)90524-9.
2. Van Buuren, S. & Groothuis-Oudshoorn, K. (2011). "mice: Multivariate Imputation by Chained Equations in R." J. Statist. Softw. **45**, 1-67.
3. Stekhoven, D. J. & Buehlmann, P. (2012). "MissForest - nonparametric missing value imputation for mixed-type data." Bioinformatics **28**, 112-118, doi: 10.1093/bioinformatics/btr597.
